# Life Conditions during COVID-19 Lockdown and Mental Health in Spanish Adolescents

**DOI:** 10.3390/ijerph17197327

**Published:** 2020-10-07

**Authors:** Lourdes Ezpeleta, José Blas Navarro, Núria de la Osa, Esther Trepat, Eva Penelo

**Affiliations:** 1 Unitat d’Epidemiologia i de Diagnòstic en Psicopatologia del Desenvolupament, 08193 Bellaterra, Barcelona, Spain; joseblas.navarro@uab.cat (J.B.N.); nuria.delaosa@uab.cat (N.d.l.O.); esther.trepat@uab.cat (E.T.); eva.penelo@uab.cat (E.P.); 2 Departament de Psicologia Clínica i de la Salut, Universitat Autònoma de Barcelona, 08193 Bellaterra, Barcelona, Spain; 3 Departament de Psicobiologia i Metodologia de les Ciències de la Salut, Universitat Autònoma de Barcelona, 08193 Bellaterra, Barcelona, Spain

**Keywords:** adolescents, COVID-19, life conditions, lockdown, mental health

## Abstract

Spanish children were locked down for 72 days due to COVID-19, causing severe disruption to their normal life. The threat posed by COVID-19 continues and clinicians, administrators, and families need to know the life conditions associated with more psychological problems to modify them and minimize their effect on mental health. The goal was to study the life conditions of adolescents during lockdown and their association with psychological problems. A total of 226 parents of 117 girls and 109 boys (mean age: 13.9; Standard deviation: 0.28) from the community that were participants in a longitudinal study answered an online questionnaire about life conditions during lockdown and the Strengths and Difficulties Questionnaire (SDQ). Stepwise regression analyses controlling by previous reports of SDQ were performed. Conduct, peer, prosocial, and total problems scores increased after lockdown. After adjusting for previous measures of psychopathology, worse adolescents’ mental health during COVID-19 lockdown was associated with unhealthy activities, worsening of the relationships with others, and dysfunctional parenting style. It seems important to mitigate psychological stress in a situation of isolation due to a state of emergency by keeping the adolescent active and maintaining their daily habits and routines in a non-conflictive atmosphere and give support to parents.

## 1. Introduction

The World Health Organization declared the emergency situation caused by COVID-19 as a global pandemic on 11 March 2020. In Spain, the nationwide state of emergency due to COVID-19 was pronounced on 14 March. The government declared lockdown, starting on 13 March and ending on 24 May. The state of emergency lasted for 72 days, during which time the population’s free circulation was limited, non-essential services/activities were cancelled, and face-to-face school teaching was suspended with the aim of minimizing the transmission of the virus through social contact. The population was called to stay at home. After 44 days of confinement, children under the age of 14 were allowed to go out for a walk for 1 h a day. Informal get-togethers with family and friends were permitted if a physical distance was maintained, while organized meetings and physical contact remained prohibited. After the lockdown period ended, several phases of de-escalation of the restrictive measures were applied until the state of emergency ended on 21 June and the population returned to new normal life, but without schools re-opening. Children experienced an abrupt disruption to their normal life and prolonged isolation from their peers and loved ones, which may have led to an increase in mental health problems [1]. Additionally, they had to cope with the psychological distress caused by the threat of the pandemic and fear of contamination. 

The epidemic extended progressively across the world and different countries were affected at various times. The short time that has elapsed since the beginning of the epidemic has meant that empirical studies about children’s mental health during the COVID-19 pandemic are scarce. Existing research has come mostly from China, where the epidemic started. Some surveys carried out using community samples report on children’s mental health during lockdown. Zhou et al. [2] found a prevalence of depression and anxiety of 43.7% and 37.4%, respectively, in 12 to 18-year-old Chinese adolescents (*N* = 8079). Being older, female, from a rural area, and having limited information about the disease increased the risk of higher scores for depression and anxiety. Xie et al. [3] reported prevalence of depression and anxiety of 22.6% and 18.9%, respectively, in 1784 Chinese students from 2nd to 6th grade when they had been locked down for 30 days of the two-month lockdown. Not being worried about COVID-19 and being optimistic were associated with lower scores for depression. Demographic variables and attitudes toward disease were not associated with anxiety scores. Jiao et al. [4] described a sample of 320 3 to 18-year-old children in China, whose parents reported that their children were clingy (37%), distracted (32%), irritable (31%), and worried (29%), they obsessively requested updates (28%), they feared for the health of relatives and had sleeping difficulties (21%), and they had a poor appetite (18%). Media entertainment was the most effective way to relieve distress, scoring higher than reading or physical exercise. Younger children presented more symptoms than older ones. In India, children isolated in quarantine experienced greater psychological distress than non-quarantined children, most commonly experiencing worry (68.6%), helplessness (66.1%), and fear (62%) [5].

In samples of children with previous mental illnesses in other countries, their conditions have been reported to be negatively affected by the pandemic [6]. This is the case of children with attention-deficit/hyperactivity disorder [7] and autism [8]. The variables that were more predictive of already existing conditions worsening were children’s and parents’ general worse mood, less time dedicated to studying [7], and not receiving support from the school [8]. Older age and living with a single parent were protection factors against more intense behavior problems in autistic children [8].

By 28 June, at the end of the lockdown, 28,363 people had died in Spain, and around 5666 had died in Catalonia, the area where the present study was carried out [9]. The COVID-19 pandemic is a stressful and traumatic event for many people. The first seroprevalence data indicate that an estimated 5.2% of the population has been infected [10], pointing to a low rate of immunization. A vaccine is being developed, but meanwhile health authorities have warned of a high risk of new outbreaks. Consequently, the feeling of threat and the state of stress are ongoing for many people. In this context, we need to know what life conditions during lockdown are associated with mental health problems in children to be able to target them in the case of new isolation measures or quarantine, thus preventing negative emotional and behavioral outcomes. Additionally, the scientific community needs to know about the mental status of the population during the lockdown period to subsequently study the probable long-term consequences. The goal of the study was to describe the life conditions of a sample of adolescents from Barcelona (Spain) during lockdown and to identify which of them are associated with mental health problems.

## 2. Method

### 2.1. Participants

The families taking part in a longitudinal study about developmental trajectories of psychological problems that recruits information every year since the children were three years-old [11] were invited to participate. When COVID pandemic started the study was in the 10th follow-up (*N* = 411) and this sample was invited to participate in COVID-19 survey. A total of 226 parents (35 fathers and 191 mothers; 55%) answered the questionnaires about 117 girls and 109 boys (mean age: 13.9; standard deviation (SD): 0.28). Of the sample, 30.1% belonged to a high socioeconomic level (SES), 54.5% to middle, and 15.4% to low. They were mainly Caucasian (92.9%), while 3.6% were American-Hispanic, and 3.5% belonged to other ethnicities. They attended to a total of 74 schools spread across Barcelona area (66.4% public education centers and 33.6% to semi-subsidized schools). Of those participating in COVID-19 survey, 197 had also participated in a 9th follow-up (age 12). Regarding the initial sample at the first follow-up, there were not differences between participants and non-participants in sex (*p* = 0.353) but attrition was higher among those in lower SES level (*p* = 0.003).

### 2.2. Instruments

Questionnaire about lockdown. A questionnaire for parents was designed to be answered online by mobile phone, or on the computer. The questionnaire included a total of 57 yes/no response format questions referring to lockdown: physical environment (12 questions), COVID-19 disease (nine questions), the adults sharing the house (11 questions), the adolescents’ relationships (three questions), the adolescents’ activities (14 questions), and the adolescents’ feelings/behaviors (eight questions). 

The Strengths and Difficulties Questionnaire (SDQ) [12] assesses children’s mental health with 25 items and a 3-point ordered response format ranging from 0 (*not true*) to 2 (*certainly true*) on five scales (in brackets ordinal alpha internal consistency): emotional problems (0.82), conduct problems (0.79), hyperactivity/inattention (0.83), peer relationship problems (0.71), and prosocial behavior (0.67). The items on the first four scales provide a total difficulties score (0.89). The questionnaire has demonstrated acceptable concurrent and discriminating validity in Spanish samples [13]. The questionnaire was completed by the parents after lockdown (named afterwards post-COVID-19) and the previous year, when adolescents were 12 years-old (named afterwards pre-COVID-19). 

### 2.3. Procedure

On 9 June, the families participating in a study about developmental psychology received an e-mail inviting them to answer the questionnaires, highlighting the importance of knowing how the participating youth had coped during the lockdown situation between 13 March and 24 May. The e-mail was sent to the children’s guardians with a link containing the instructions and the deadline date. A second mail with the link to the questionnaires and the password to complete them was also sent. The questionnaire about lockdown was presented first, followed by the SDQ. The main project was approved by the Ethics Committee on Animal and Human Experimentation of the Universitat Autònoma de Barcelona (CEAAH 4324).

### 2.4. Statistical Analysis

The data analysis was carried out using Stata 16 (Stata Corp LLC, College Station, TX, USA). Because the multistage sampling designed for the longitudinal study gave unequal probabilities of being selected, analyses were weighted by the inverse of the probability of participants’ being selected in the second phase of the sampling (see [11]).

The means pre–post COVID-19 for each SDQ scale plus the total were compared through paired *t*-test. Hedges’ *g* effect sizes for paired samples [14] was calculated for each significant class comparison; absolute values were interpreted as: null effect for values <0.20, small effect for values 0.20–0.50, medium effect for values 0.50–0.80 and large effect for values >0.80 [15].

The association between the set of items in each category of life conditions during lockdown and the post-COVID-19 SDQ-scores was calculated using forward stepwise linear regression analysis (*p*_enter_ = 0.050; *p*_remove_ = 0.051), including the SDQ pre-COVID-19 measure as a fixed adjusting term. Given that predictors were binary, *b* represents the mean difference in SDQ post-COVID-19 between the presence/absence of each life condition adjusted by the other variables in the same category. *R*^2^ shows the percentage of SDQ scores explained by the model. The effect size for linear regression was measured by Cohen’s *f*^2^, interpreted as null for values <0.02, small for values 0.02–0.14, medium for values 0.15–0.34 and large for values >0.34 [16]. *R*^2^ and *f*^2^ were calculated excluding the adjustment SDQ pre-COVID-19 score from the model with the set of selected life condition predictors.

### 2.5. Data Availability Statement 

Data cannot be made publicly available due to ethical restrictions protecting the confidentiality of the families involved. Data are available to interested researchers after signing consent of confidentiality form as the authors had previously signed to obtain the data from the families of the sample. Researchers must be working in clinical child psychology in a public funded project. To request the anonymous data, please contact the corresponding author.

## 3. Results

### 3.1. Life Conditions

**Figure 1:**
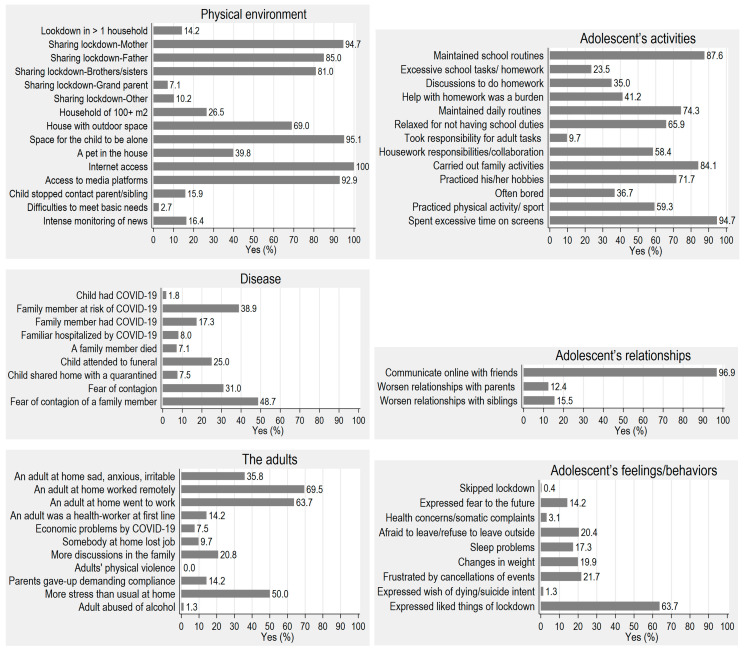
Percentage of affirmative response for each group of COVID-19 questions (*N* = 226).

Figure 1 shows the distribution of the different areas of life conditions. Regarding the physical environment, during lockdown the adolescents stayed in one household (>80 m^2^), with a mean of four people (mother, father, siblings) and had access to the Internet and media entertainment platforms. Over a third of the families were affected in some way by COVID-19 (suffered the disease, hospitalized, quarantined, or died) or feared contagion. About two thirds of the adults continued to work (remotely or went to work), while around 10% lost their jobs and had financial difficulties. Half the adults reported that there was more stress than usual at home and one third felt sad, anxious, hopeless, or irritable, and one tenth gave up demanding compliance. The adolescents kept up online communication with friends, and the relationships with their parents and siblings worsened in about 10% of cases. Most of the adolescents kept up their schoolwork and daily routines, felt relieved because there was less schoolwork, got involved with family activities, did their hobbies, and definitely spent more time than usual on screens. However, for about one third, the amount of schoolwork was excessive and completing it caused the family increased stress. Half the adolescents helped with the housework and 10% took on adult responsibilities (the care of siblings, other dependents, or sick people). About one fifth of the adolescents expressed some negative feelings (frustration, fear of leaving the house, fear of the future), had sleeping difficulties, or had weight changes. Two thirds recognized that there were some good things about lockdown. 

### 3.2. Change in Psychological Symptoms Pre and Post COVID-19

Table 1 presents the means of the SDQ scale scores pre-COVID-19 pandemic, obtained at the 9th follow-up of the study when adolescents were 12 years old, and post-COVID-19 lockdown, obtained when adolescents were 13 years old. Psychological symptoms increased significantly after the lockdown in the areas of conduct problems, peer problems, prosocial behavior, and total difficulties, decreased in emotional problems and did not change in hyperactivity–inattention problems. Effect sizes were small for emotional, conduct and total problems (0.14–0.27) and medium for peer and prosocial behavior problems (0.61–0.79).

### 3.3. Association between Life Conditions and Mental Health

Table 2 presents the significant association between life conditions in each category and post-COVID-19 psychopathology (SDQ scale scores), adjusting for pre-COVID-19 psychopathology. Higher emotional problems scores were mainly associated with the adolescents’ feelings and behavior during lockdown (excessive concerns about health, sleep problems, feelings of frustration about cancellations; 26.2% of common variance), followed by what the adolescent’s relationships were like (worsening relationships with their parents; 17%), the adolescents’ activities (parents overburdened with helping with homework, frequent boredom; 13.4%), how the adults in the household behaved (more stress in the family; 11.8%), and concern about contagion (7.7%). Conditions of the physical environment were not associated with emotional problems. Effect sizes ranged from small (0.08) to large (0.36).

Higher conduct problems scores were mainly associated with adolescents’ relationships (worsening relationships with family members and not communicating online with friends; 29.9% of common variance), followed by how the adults in the household behaved (more discussions in the family, giving up enforcing the rules; 25.4%), and the adolescents’ activities (not keeping up with schoolwork or daily routines, excessive time on screens; 23.3%). Sharing lockdown with a person in quarantine decreased conduct problems. Adolescents’ feelings/behavior during lockdown and the physical environment were not associated with conduct problems. Effect sizes ranged from small (0.01) to large (0.43).

Higher hyperactivity–inattention problems scores were mostly associated with the adolescents’ activities (having few school work, arguments about homework, not keeping up with daily routines; 20.7% of common variance), followed by the adolescents’ relationship with their parents (worsening; 9.3%), the adults’ behaviors (more discussions, lack of limit setting; 6.9%), the physical environment variables (being locked down with people who were not immediate family or with siblings; 4.6%), and the adolescents’ feelings/behaviors (concerns about health, not being afraid of going outside, sleep problems; 3.9%). How the disease impacted the family was not associated with hyperactivity/attention problems. Effect sizes were small (0.04–0.10) except for adolescent’s activities (0.26).

Higher peer problems scores were associated with the adolescents’ activities (not doing joint activities with the family, boredom, not doing any physical activity, excessive time on screens; 13.2% of common variance), followed by the adolescents’ relationships (lack of online communication with friends, bad relationships with siblings in the house; 6.9%), the physical environment (not sharing lockdown with other siblings; 4.9%), and last the affectation of the disease (a person in the immediate family had COVID-19; 0.4%). Effect sizes ranged from almost null (0) to medium (0.15).

Higher prosocial problems scores were mostly associated with the behavior of the adults (parents giving up enforcing the rules and more stress than usual at home; 12.4% of common variance), followed by the adolescents’ activities (not doing joint activities with the family; 5.2%), and the adolescents’ feelings (not expressing fear to the future, presenting somatic complaints, refusing to go outside; 5%). Effect sizes were small (0.05–0.14).

Higher total problems scores were associated with the adolescents’ activities (not keeping up daily routines, parents overburdened with helping with homework, not doing joint activities with the family, boredom, excessive screen time; 26.2% of common variance), followed by the adolescents’ relationships (lack of online communication with friends, worsened family relationships; 25.3%), the adults’ behavior (more discussions in the family, parents gave up enforcing the rules; 17.6%), and last changes in weight (4.3%). The physical environment and how the disease affected the family were not associated with total problems. Effect sizes ranged from small (0.04) to large (0.36).

## 4. Discussion

Controlling for previous measures of psychological problems, Spanish adolescents’ mental health during the COVID-19 lockdown was associated most importantly with characteristics to do with the activities the adolescent kept up, with the quality of their relationships, with how the adults were affected by the lockdown, and, to a lesser extent, with the physical environment where they were locked down, with how they reacted to the lockdown in terms of feelings and behaviors, and with how the disease affected the immediate family. The associations found may be informative: (a) to advise the population in the case of new outbreaks or quarantine about the optimal conditions to minimize the effects on the whole lockdown unit; (b) to inform clinicians about the potential risk conditions for their clients in the case of new outbreaks; (c) to select target conditions that must be modified or improved in the different contexts (school, family, health system, etc.) in the case of outbreaks in order to minimize impacts; and (d) to inform the design intervention guides for specific mental health risk groups.

Conduct, peer, prosocial, and total problems significantly increased post-COVID-19 lockdown in comparison with measures previously obtained, meaning that lockdown may have had an effect on the increment of the problems. Hyperactivity–inattention, a chronic neurodevelopmental disorder, remained stable and did not change pre and post-COVID lockdown. Notably, emotional problems, as evaluated by parents, decreased after the lockdown. This unexpected decrease may be related with the fact that the informants were the parents, and parents are not the best informants of the emotional state of the children [17], which highlights the need to obtain emotional information from the adolescents. Clinicians, researchers, educators, and families will need to surveil future evolutions of the increased symptomatology.

Regarding the psychological problems evaluated, the variables that were related to the widest variety of problems were a worsening of the adolescents’ relationships, a parenting style characterized by lack of limit setting, together with an increase of discussions in the family, the lack of online communication with friends, not doing joint activities with the family, feelings of boredom, and an increase of concerns about health. Most of these variables reflect how difficult family relationships may be for adolescents and their families living together closely under stress during a lockdown and the importance of keeping activities and relationships with peers. A special mention needs to be made of the excessive use of screens, which was reported by 95% of the parents. Although access to media entertainment platforms may be protective during a lockdown, as shown in Chinese samples [4], excessive screen time may be associated with increased conduct, peer and total problems, therefore requiring proper dosage. Also, changes in important physiological functions, such as eating and sleeping, merit some comment, as they affected about a 20% of the sample. When children are not at school, for example at the weekend or in the holidays, they tend to change their routines and are usually less physically active, they spend more time on screens, and their sleep patterns and diets are altered, resulting in weight gain [18]. It therefore seems important to mitigate the psychological difficulties faced in a situation of long isolation due to a state of emergency by keeping the adolescent active and keeping up their daily routines, including hygiene habits and schedules for waking-up, eating, sleeping, and so on, in a non-conflictive family environment. Furthermore, schools should carefully monitor the amount of online work they assign to ensure that it is not excessive, providing parents with guidelines on how to supervise the tasks. For 40% of the parents help with homework was overburdening them.

Stress is one of the main risk factors of suicide [19]. The enormous stress posed by the COVID-19 lockdown on adolescents and their families, reflected in part in the parents’ observations of their adolescents’ wish to die, must be highlighted. We do not yet know how COVID-19 will affect suicide rates [19]. As expected for a general population sample, few adolescents expressed these feelings (*N* = 3; 1.3%) but still this rate need to be noted due to their clinical relevance.

Emotional problems were associated with fear of contagion. As has been reported in Chinese children [20], fear and worry about themselves contracting the disease was related to higher scores in emotional difficulties. Similarly, situations related to emotionality (conflicts and stress in the family, feeling overwhelmed, frustration, concerns about health) and mood (boredom) were associated with higher emotional problems. As would be expected, emotional problems were related to sleep problems [21]. Emotional problems shared most variance with adolescent’s feelings/behaviors variables (26.2%). Therefore, to minimize the effects of lockdown on emotional symptoms, it is advisable to promote the emotional self-control of the whole unit, to get a good balance between analysis of the problem (real threat) and coping, and to have good sleep hygiene. Communicating with the adolescent about life-threatening illnesses in a sensitive, effective way has the best long-term benefits for them and their families [22].

Meanwhile, the observed increase in conduct problems, which are typically characterized by rule breaking, was related to conflict in the relationships with family members, dysfunctional parenting, not following routines, and spending excessive time on screens. Notably, sharing lockdown with a person in quarantine was associated with lower scores in conduct problems. This finding may have different interpretations, one of them being that, in such a situation, adolescents collaborate with the family and presented a proper behavior. Alternatively, it could be that the adults were more centered in the family member in quarantine and relativize behavior problems. Nonetheless, hyperactivity–inattention problems shared most variance with the adolescents’ activities, including struggling to complete homework and keeping up with daily routines, in addition to increased conflict with parents and dysfunctional parenting practices. Accordingly, to minimize the effects of lockdown on conduct problems and hyperactivity–inattention it is advisable to maintain previous routines (including sleep patterns) and to provide parental support to enforce the rules in a lockdown situation. In this line, several world organizations have provided free parenting resources to build positive relationships, manage bad behavior, and manage parenting stress (see [23]).

Although the adolescents were locked down, 97% of them maintained online communication with their peers. Increased difficulties with peers were associated with lack of activity (with the family, physical activity, boredom, not communicating online with friends) and excessive activity on screens. However, peer problems shared little variance with the studied variables and the few associations found may reflect stressful situations that might have interfered with maintaining contact with peers (family member with COVID-19) and that lockdown impaired relationships with equals (other siblings and peers). Prosocial behaviors also shared low common variance with lockdown life conditions. The highest shared variance was with dysfunctional parenting and stress in the family. In an emergency situation it is crucial that all individuals help each other and are empathetic, including in the family nucleus. Parents should strengthen their adolescents’ prosocial behavior, which may be crucial to achieving their collaboration regarding preventative measures, such as respecting physical distances and using a face mask [24].

To our knowledge this is the first study on life conditions during the 72-day COVID-19 lockdown in Spanish adolescents and their associations with psychological problems measured using a well-recognized assessment instrument as reported by parents and controlling for previous psychological problems. Some limitations should be considered when interpreting the results. The study was carried out on a non-probabilistic sample of volunteers in a cohort already being followed. The cross-sectional design prevents the directionality of the associations from being established. The study reports the concurrent association between life conditions and psychopathology restricted to the lockdown period as observed by parents and controlling by previous measures of psychological problems. There were few participants in the lower socioeconomic levels, so consequently the results cannot be generalized to the most disadvantaged groups. Future research should describe the longitudinal impact of lockdown on mental health.

## 5. Conclusions

After lockdown, and controlling by previous measures of psychopathology, adolescent’s conduct, peer, prosocial, and total problems scores increased significantly, with effect sizes that ranged from small to medium. 

Most of the adolescents spent lockdown in physical environment that covered their main needs, but about half of the adults reported that the family was living more stress than usual. Affectation by the disease, lost of job and financial difficulties, increase of family discussions and mood variations were some of them. The adolescents kept up online communication with friends and their schoolwork and daily routines and they spent more time than usual on screens. However, for a part of them, the relationships with their parents and siblings worsened, the amount of schoolwork was excessive, and some expressed some negative feelings, had sleeping difficulties, or had weight changes. Two thirds recognized that there were some good things about lockdown. 

After adjusting for previous measures of psychopathology, worse adolescents’ mental health during COVID-19 lockdown was associated with unhealthy activities, worsening of the relationships with others, and dysfunctional parenting style. It seems important to mitigate psychological stress in a situation of isolation due to a state of emergency or quarantine by keeping the adolescent active and maintaining their daily habits and routines in a non-conflictive atmosphere, and give support to parents.

## Figures and Tables

**Table 1:** Change in the Strengths and Difficulties Questionnaire (SDQ) psychological problems scores between Pre and Post COVID-19 (*N* = 197).

SDQ Scale	Pre-COVID M (SD)	Post-COVID M (SD)	Difference (CI 95%)	*|t|*	*p*	*g*
Emotional problems	1.21 (1.57)	0.82 (1.26)	−0.40 (−0.62; −0.16)	3.40	<0.001	0.27
Conduct problems	0.98 (1.28)	1.23 (1.33)	0.25 (0.07; 0.42)	2.80	0.006	0.19
Hyperactivity–inattention problems	2.49 (2.40)	2.50 (2.11)	0.01 (−0.23; 0.26)	0.12	0.902	
Peer problems	0.77 (1.29)	1.65 (1.56)	0.88 (0.66; 1.09)	8.04	<0.001	0.61
Prosocial behavior problems	1.46 (1.57)	2.85 (1.93)	1.40 (1.13; 1.67)	10.21	<0.001	0.79
Total difficulties	5.45 (4.65)	6.20 (4.44)	0.75 (0.22; 1.28)	2.81	0.005	0.14

M: mean; SD: standard deviation; CI: confidence interval; g: Edge’s effect size.

**Table 2:** Life condition predictors of post-COVID-19 psychopathology (*N* = 197).

SDQ Scale	Emotional Problems			Conduct Problems			Hyperactivity–Inattention Problems			Peer Problems			Prosocial Behavior Problems			Total Difficulties		
	*b*	*SE*	*p*	*b*	*SE*	*p*	*b*	*SE*	*p*	*b*	*SE*	*p*	*b*	*SE*	*p*	*b*	*SE*	*p*
**Physical environment**																		
People sharing lockdown-Other							1.13	0.49	0.023									
People sharing lockdown- brothers/sisters							1.13	0.49	0.023	−0.81	0.32	0.013						
*R*^2^ (Cohen’s *f*^2^):							4.6% (0.05)			4.9% (0.05)								
**Disease**																		
Shared home with person in quarantine				−0.85	0.31	0.007												
Person in immediate family had COVID-19										0.50	0.24	0.039						
Fear of contagion	0.65	0.21	0.002															
*R*^2^ (Cohen’s *f*^2^):	7.7% (0.08)			0.8% (0.01)						0.4% (0.00)								
**The adults**																		
More discussions in the family				0.80	0.26	0.002	0.86	0.27	0.002							2.33	0.73	0.002
Parents gave-up setting limits				1.36	0.25	<0.001	0.72	0.37	0.050				1.13	0.44	0.012	2.88	0.92	0.002
More stress than usual at home	0.74	0.19	<0.001										0.50	0.25	0.045			
*R*^2^ (Cohen’s *f*^2^):	11.8% (0.13)			25.4% (0.34)			6.9% (0.07)						12.4% (0.14)			17.6% (0.21)		
**Adolescent’s relationships**																		
Communicate online with friends				−0.98	0.27	<0.001				−1.28	0.59	0.033				−2.86	1.10	0.010
Worsened relationships with parents	1.36	0.40	0.001	1.11	0.29	<0.001	1.23	0.41	0.003							3.54	0.95	<0.001
Worsened relationships with brothers/sisters				0.87	0.30	0.004				0.61	0.27	<0.001				2.13	0.77	0.006
*R*^2^ (Cohen’s *f*^2^):	17.0% (0.20)			29.9% (0.43)			9.3% (0.10)			6.9% (0.07)						25.3% (0.34)		
**Adolescent’s activities**																		
Maintained school routines																−2.75	0.96	0.005
Excessive school tasks/homework							−0.69	0.25	0.005									
Discussions/tension to do homework				0.75	0.21	<0.001	0.87	0.27	0.001									
Parents help with homework was a burden	0.49	0.18	0.008													1.30	0.54	0.017
Maintained daily routines				−0.52	0.25	0.041	−0.95	0.44	0.032									
Carried out family activities										−0.93	0.31	0.003	−1.24	0.37	0.001	−1.84	0.92	0.047
Often bored	0.63	0.21	0.003							0.43	0.21	0.039				1.57	0.57	0.007
Practiced any physical activity/sport										−0.43	0.21	0.040						
Spent excessive time on screens				0.71	0.16	<0.001				0.75	0.28	0.008				2.29	0.56	<0.001
*R*^2^ (Cohen’s *f*^2^):	13.4% (0.15)			23.3% (0.30)			20.7% (0.26)			13.2% (0.15)			5.2% (0.05)			26.2% (0.36)		
**Adolescent’s feelings/behaviors**																		
Expressed fear to the future													−0.93	0.34	0.007			
Concerns health/somatic complaints	1.38	0.57	0.016				0.94	0.45	0.038				1.07	0.50	0.032			
Afraid to leave home/refuse to leave							−0.91	0.24	<0.001				0.72	0.29	0.015			
Sleep problems	1.16	0.41	0.005				0.68	0.30	0.023									
Changes in weight																1.69	0.70	0.017
Frustrated by important cancella tions	0.58	0.27	0.035															
*R*^2^ (Cohen’s *f*^2^):	26.2% (0.36)						3.9% (0.04)						5% (0.05)			4.3% (0.04)		

*b*: Regression coefficient; *SE*: Standard error; *R*^2^: percentage of common variance between life conditions and SDQ scores; *f*^2^: Cohen’s effect size. Estimations adjusted by SDQ pre-COVID-19; *R*^2^ and Cohen’s *f*^2^ values calculated without SDQ pre-COVID-19 as predictor.
